# Intersectionality as a theoretical framework for researching health inequities in chronic pain

**DOI:** 10.1177/20494637231188583

**Published:** 2023-07-09

**Authors:** Cassandra Macgregor, Jackie Walumbe, Emmanuelle Tulle, Christopher Seenan, David N Blane

**Affiliations:** 1 School of Health and Life Sciences, Glasgow Caledonian University, Glasgow, UK; 2 NHS Lanarkshire Chronic Pain Service, Buchanan Centre, Coatbridge, UK; 3 Nuffield Department of Primary Care Health Sciences, University of Oxford, Oxford, UK; 4 University College London Hospitals NHS Foundation Trust, London, UK; 5 Department of Social Sciences, Glasgow Caledonian University, Glasgow, UK; 6 School of Health and Wellbeing, University of Glasgow, Glasgow, UK

**Keywords:** intersectionality, health inequalities, chronic pain, theory, epistemology, pain care, equity, health inequities

## Abstract

Chronic pain is experienced unequally by different population groups; we outline examples from the pain literature of inequities related to gender, ethnicity, socioeconomic and migration status. Health inequities are systematic, avoidable and unfair differences in health outcomes between groups of people, with the fundamental ‘causes of causes’ recognised as unequal distribution of income, power and wealth. Intersectionality can add further theory to health inequities literature; collective social identities including class/socioeconomic status, race/ethnicity, gender, migration status, age, sexuality and disabled status intersect in multiple interconnected systems of power leading to differing experiences of privilege and oppression which can be understood as axes of health inequities. The process of knowledge creation in pain research is shaped by these interconnected systems of power, and may perpetuate inequities in pain care as it is largely based on majority white, middle class, Eurocentric populations. Intersectionality can inform research epistemology (ways of knowing), priorities, methodology and methods. We give examples from the literature where intersectionality has informed a justice oriented approach across different research methods and we offer suggestions for further development. The use of a reductionist frame can force unachievable objectivity on to complex health concepts, and we note increasing realisation in the field of the need to understand the individuals within their social world, and recognise the fluid and contextual nature of this.

## Introduction

Intersectionality is of increasing interest to the research community in the field of chronic pain, and aspects of intersecting health inequities have recently appeared in this journal.^1,2^ We add to the conceptual framing of this emerging area by providing an introduction to key concepts and an outline of intersecting axes of health inequities in the experience of chronic pain and pain care. We then explain how and why intersectionality provides a relevant and useful theoretical framework to inform justice-orientated research in chronic pain and pain care. We provide examples from the literature of how intersectionality has been, and could be, used in different ways in researching health inequities, chronic pain and pain care, at the epistemological and methodological levels (including literature review, qualitative and quantitative designs), with suggestions for future work and development.

## Introduction to health inequities and the context of this paper

Health inequities are ‘systematic, avoidable and unfair differences’ in health outcomes between groups of people.^3^ The fundamental ‘causes of causes’ are an unequal distribution of income, power and wealth reflecting wider socioeconomic inequality.^4^ Health inequities can be conceptualised as social, political and economic determinants of health,^5^ with both ‘upstream’ and ‘downstream’ components. The ‘upstream’ factors include policies governing access to education, reproductive rights, housing, secure employment, de/regulation and marketisation of healthcare. These, in turn, flow ‘downstream’ to the ‘effects of causes’, including individual behaviours, lifestyle and biological factors.^5,6^ The framing of health inequities as related to structural inequities is important to a population level understanding of health. Countries with higher income inequality tend to have worse population health outcomes than others with comparable income levels; high levels of income inequality are bad for all of us and narrowing the gap can improve population health.^7^

With ageing populations and increasing burden of non-communicable diseases such as back pain, headache, mental health disorders and opioid addiction, inequities in the experience of these conditions and available care is of global concern.^8^ The context of the intersecting axes of health inequities will vary at a national level due to different population compositions, historical and cultural systems.^9^ In this paper, we mainly, but not exclusively, discuss intersectional health inequities in the context of high-income countries with majority white populations. These are either European countries, or countries colonised by Europeans including the U.S., Canada, South Africa and Australia.

We note variation in terminology applied to health inequities. ‘Inequalities’ and ‘inequities’ are often used interchangeably in research and policy literature. We use the terms equity and inequities, relating to the justice elements of the term and its use internationally, and the definition ‘the systematic, avoidable and unfair differences in health outcomes that can be observed between populations, between social groups within the same population’.^3^ A ‘health disparity’ is a ‘difference in a measurement of a health variable comparing more than one individual or group with specific defining characteristics, after controlling for individual health choices, different disease courses, variation from the norm and genetic factors’,^10^ without necessarily including the systematic and unjust element of the concept. We do not use the term health disparity further in this paper as we focus on inequities and equity. We use the plural, ‘inequities’, to acknowledge their multiple and intersecting nature.

Power is key to the theoretical framing of health inequities, bringing in analysis of political and cultural systems and ideologies, and social capital and connections, which influence an individual’s capacity for health and wellbeing.^4,11^ Power is an insidious, invisible concept, the impact of which is often more apparent to those who hold less power and may experience the resulting barriers, for example, in the healthcare provider/patient relationship.^4,11,12^ Intersectionality provides a broad theoretical framework to conceptualise the role of power in health inequities.

We advocate for researchers to consider power in many ways and this starts with considering positionality, which means ways of understanding our own social position in relation to the research.^13^ We do so in order to consider how we ourselves may influence our research, and reflect on what this means for our role in this particular piece of knowledge (being reflexive). To be explicit about our own positionality, CM and JW are cisgender, straight and non-disabled women. In reference to self-identified ethnicity, CM is white and Scottish and JW is black and African. ET is a woman of mixed parentage, white European and African. CS and DNB are white, Scottish men, who are straight, cisgender and non-disabled. We are all of professional middle class background with academic roles ranging from university professor to PhD student. Three of us work clinically and provide care for people with chronic pain, many of whom may be implicitly, or explicitly, excluded from research. We do not have lived experience of chronic pain; we do have experience of clinical application of research, and of the myriad choices, time and effort involved in the research process. All of us live in the U.K., mostly Scotland.

## Introduction to chronic pain

Chronic pain is a heterogeneous category of conditions, recognised as either a primary condition itself, symptom of a secondary condition where the pain has persisted for over 3 months, or both.^14^ Pain itself is a complex and contested phenomenon, broadly considered to be subjective in nature, and a result of interpretation by the person experiencing it within a cultural context.^15,16^ Chronic pain often defies specific, structural diagnosis, bringing the notion of subjectivity into conflict with that of ‘objectivity’, leading to issues of trust between provider and patient, and lack of fit with dominant biomedical models and systems.^17^ This context can heighten the impact of societal norms, identities, biases and ideologies on the person with chronic pain.^16–18^ Therefore, our analysis of this context can further benefit from ideas from sociology, including intersectionality.^16,18,19^

The social, political and economic determinants of health cause distress and can lead to poor mental health, which is unevenly distributed across the population and is a key mechanism of health inequities.^4,20,21^ The contribution of distress to chronicity of pain is well established and accepted^15,22–24^ and this includes adverse childhood experiences^25^ and different forms of oppression.^18,26^ Racism and the gendered nature of violence and trauma can add to more established social determinants of health, with distress negatively impacting on health via multiple biological mechanisms. ^27^

Chronic pain is a long-term condition, typically experienced alongside other comorbidities, and therefore brings condition management ‘work’, which can include coordination and execution of tasks associated with managing the condition, emotional management and motivation, negotiation and coordination with healthcare, and biographical work (navigating a challenge to identity and the contrast with life before chronic pain).^28,29^ The capacity required for condition management work can be impacted by health inequities, including wealth, power, income, access, education, social connection and the prevailing political ideological approach to benefits and healthcare.

## Introduction to intersectionality

**Figure 1.**
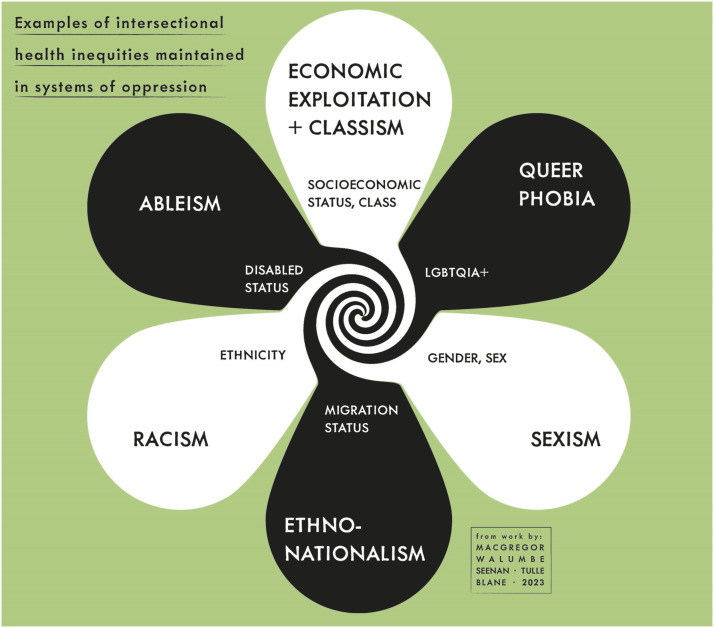
Examples of contextual intersectional health inequities.

Intersectionality posits that collective social identities of class/socioeconomic status, race/ethnicity, gender, ‘nation’, sexuality and disabled status intersect in multiple interconnected systems of power leading to differing experiences of privilege and oppression which can be understood as axes of health inequities.^6,30,31^ Intersectionality developed from black feminist thought in the United States, from as early as the 19^th^ century, through the activist-scholars of the 1970s, to a current established base in sociology and health literature.^6,31–33^ Crenshaw coined the term in a legal text, arguing that black women faced multiple discrimination because they were *both* women and black.^34^ Intersectionality has developed from the core ‘race, class, gender’ framing to a typically wider range of intersections which are culturally and context specific.^30^ Intersections can further extend into age, disabled status, sexuality, trans status and the power differentials associated with ‘nation’,^30,33^ explored with immigration status in chronic pain inequities research.^1,19^ Intersectionality maintains the importance of class and socioeconomic status, yet offers an extension from its historical dominance in health inequities research in the UK and Europe.^30,35^ Categorisations of social identity should be understood as fluid, dynamic, created and maintained by societal systems of power; the latter should be given more focus in health inequities research.^35^ Individuals live within a society which is, to differing contextual degrees, gendered, racialized, classist and ableist, and these axes of privilege/oppression lead to inequities in the experience of chronic pain. This conceptualisation is illustrated in Figure 1.^18,19,36^

Inequities in the experience of chronic pain and pain care are intersectional in nature and we briefly outline examples of these in Table 1.

**Table 1.** Examples of intersectional health inequities in chronic pain.

Socioeconomic inequities
• Chronic pain has higher prevalence, severity and is experienced as more disabling with groups ascribed lower socioeconomic status and in areas of deprivation.^72,73^
• Higher levels of opioid prescribing exist in areas of deprivation.^74,75^
• Inverse care Law: Lower levels of services relative to need in areas of deprivation.^2,76,77^
• Pain commonly coexists with multi-morbidity, where combined physical and mental health conditions vary two to three-fold with increasing deprivation.^78^
Gender/sex
• Women experience higher prevalence and severity of chronic pain than men.^18,73,79^
• Currently, differences can be partly explained by sex inequities (biological) and gender inequities (socially constructed) in chronic pain experiences.^80^
Ethnicity
• In the US and UK, chronic pain has higher prevalence within racially minoritised groups, including 10% higher prevalence in black groups relative to all other ethnic groups in England. ^27,73,81^
• Ethnic categories are complex, historical, political and change over time, with racism increasingly recognised as a mechanism in ethnic health inequities which influences social determinants of health such as quality education, access to healthcare and the labour market.^35^
• Stress associated with racism can manifest biologically, with mechanisms including: Provider bias, differences in prescribing; racial cultural stereotypes; variable access to healthcare relative to need; mistrust in the scientific process due to historical racism in scientific practices.^27,47^
• Black patients presenting with potentially life limiting sickle cell disease (SCD) crises to emergency departments in the US, experience longer waits than a general sample with comparable conditions.^82^ Under-treatment and barriers to implementing guidance for SCD in emergency departments includes (majority white) provider concerns about the opioid pandemic, addiction and implicit bias.^83^ The multi-layered situation of inadequate care for SCD, a painful condition which disproportionately affects black people, is now more fully understood through an antiracist lens.^84,85^
Migration status and nation
• Collins^30,33^ advocates the concept of ‘nation’ in intersectionality. Migration status is used in recent health literature (discussed below) and may affect access to healthcare, provider bias, and sources of distress.
• Miall et al.^59^ found variation across ethnic groups with migration status, in health inequities data from Scotland, suggesting migration status must be understood in the context of other intersections.
• Culturally and linguistically diverse communities in Brady et al.^19^ described the stigma of immigration status as, for instance, a perception they were ‘only in country for benefits’, and misperceptions between provider and patient about motivations for attending healthcare.
• A participant in an evaluation of provision for racially minoritised service users in England did not attend a pain group due to uncertainty and worry about her immigration status.^1^
• Other impacts on access to care can include language, differences in culture and prevailing models of health, and the cultural relevance of many Eurocentric pain treatments and measures.

## Challenging our ‘ways of knowing’

Intersectionality can variously be understood as a theory, theoretical framework, heuristic device, methodology, concept, paradigm, tool for action and an informed stance.^6,30,31,33^ A theoretical framework provides a lens through which to understand a body of literature and develop an approach to research, similar to providing scaffolding to a study.^37^ We advocate intersectionality as a theoretical framework for chronic pain research due to key concepts of relevance including intersecting oppressions, the epistemological relevance of lived experience in knowledge creation, power, social justice and logical connections between these concepts.

Health concepts are often neither solely subjective, nor objective; rather they are socially constructed, influenced by our cultures, systems, politics and medical practices.^38,39^ Use of a constructionist or critical lens can facilitate questioning of knowledge claims based on historical and cultural assumptions, and the inherent power relations,^39,40^ leading to deconstruction of assumed ways of knowing. In the field of chronic pain, we could improve our awareness of how common concepts are socially constructed.^41^ For example, responding to concerns at the lack of contextual awareness of the individual with pain, limited conceptualisation and associated poor terminology, Webster et al.^42^ recently deconstructed ‘catastrophising’ and its common application to pain. Analysis of patient survey responses provided evidence of poor patient care, lack of validation, and a gendered aspect of women feeling dismissed by healthcare staff. If our knowledge as socially constructed can be further attended to, we may focus on the care and cultural systems that lead to these developments. The concept of ‘catastrophising’ has some useful elements that if better managed and conceptualised (by the patient, provider, system, culture), should be retained, and understood in a fuller context.

‘Acceptance of chronic pain’ provides a further example of an important concept in pain care where focus is often given to the individual, psychological construct, but could also be understood as socially constructed, either in addition to, or as an alternative to the psychological construct. In this way, acceptance of chronic pain can be conceptualised as interdependent with: cultural interpretations of pain and illness; socioeconomic capital; availability of, and interactions within health, work and social security systems; many may struggle to negotiate a pain diagnosis and access research based multidisciplinary care.^43^ Focus on acceptance understood solely through a lens of individualism and personal choice are influenced by neoliberal ideology which has permeated health research.^6^ Through the decontextualisation of an individual factor, the person with pain is more susceptible to the social, economic and political determinants of health and oppressions associated with these. Through understanding acceptance as socially constructed, interdependent on social systems and cultural ideology, more focus may be given to improving these structures around the person in pain. Importantly, improving these structures may foster increased capacities within the individual for improved health and wellbeing while living with chronic pain.

A criticism of the process of knowledge creation in chronic pain research which may perpetuate inequities in pain care is that it is largely based on majority white, middle class, Eurocentric populations.^27,44^ We therefore know more about what pain management works best for these groups, which is then disseminated as universal in recommendations. Exclusionary elements of research include exclusion from studies of people with comorbidities (e.g. mental health problems) or language and literacy difficulties; and practical and financial barriers (e.g. transport issues) to participation, leading to inequities in the evidence cycle which then become self-perpetuating. Approaches to health which focus on individual choice and behaviours may benefit people with a higher socioeconomic status but are less likely to be effective in lower socioeconomic populations.^6,20^ We require to constantly re-evaluate issues of power, culture and the impact on our knowledge base, and capacity for health.

A recent example of this issue with knowledge creation is that of Pain Reprocessing Therapy (PRT). Ashar et al.,^45^ report that following ‘community’ recruitment, less than 15% of those assessed for online eligibility were included in the final study. Treatment group demographics (in the U.S.) were 78% college graduate, 92% white and 80% undertook three or more hours of exercise per week, and the authors acknowledge this as a limitation. The study population is not representative of the U.S. population, furthermore, given that chronic pain is experienced with more severity and prevalence across intersectional health inequities, new research developments should focus on including the groups who require to benefit the most for maximum societal benefit, with a minimum aim of being representative of the population.

If we create knowledge about pain rehabilitation and therapy with relatively privileged groups, then apply those programmes or therapeutic models to disadvantaged groups, there is a danger that they will not be ‘fit for purpose’. Rather, we should co-develop therapy programmes with the groups who experience higher levels of pain, distress and oppression. The treatment mechanisms claimed in PRT include neurobiological distress mechanisms and their interaction with the experience of pain. Therefore, the unequal experience of distress across the population, discussed earlier, should be considered in the development of PRT research and treatment.

Intersectionality can be used as a framework to centre marginalised groups in the research programme, which will require adjustments to study recruitment and data collection strategies to reach groups who may experience more poverty, racism, sexism and other forms of oppression. The distress associated with these forms of oppression could then be factored into the current development of PRT, understanding how and if treatment can benefit these groups; it is important that care targeting the individual is not decontextualized. Recruitment of those attending primary care for their pain, working with clinicians as recruiters, rather than community advertising may help achieve this. Adjustment of data collection methods, so they do not necessitate MRI scans, treatment attendance to a lower frequency than twice a week, in accessible locations, and involvement of people with lived experience in methodological development could benefit. We discuss literature on trial inclusion later in the paper.

## Intersectionality and social justice

Social justice and aims of equity lie at the core of intersectionality.^30,33,46^ The equitable aim of intersectionality is consistent with recent antiracist framework publications in the field of pain which advocate deconstruction and re-framing of research.^27,47,48^ An intersectional framework is necessary for conceptualising how health inequities in chronic pain may be reduced, given their maintenance in systems of power, driving the focus away from individual ‘downstream’ factors towards ‘upstream’ factors including political ideologies of capitalism and neoliberalism.^6,31,35^ The seminal thinkers are consistently anti-capitalist in their work due to the inequities and individualism the economic model perpetuates.^32,33,46^ Neoliberalism is criticised in the health literature for its focus on individualism, blaming the individual for poor health behaviours and ‘choices’, leading to stress and depression, rather than focussing on the more powerful social, economic and political determinants of health.^6,20^ In the field of chronic pain, the opioid crisis in the US offers a tragic example of the role of capitalism, the free market and neoliberalism leading to many deaths due to poor regulation, addiction and misuse of medications with short termism delivered by private healthcare; medicine prescription trumping interdisciplinary care.^18,49^ As a counter, a ‘wellbeing economy’ places priority on human and ecological wellbeing over economic growth with increasing recognition of the link between the systems that perpetuate poor health, inequities and climate change.^50^

## The role of ‘lived experience’ and epistemic injustice

Epistemic injustice refers to a prejudicial impact on our ways of knowing which downgrades the experience of certain people or groups, linked with systematic inequities in power.^51^ Lack of input from people who experience health inequities into research and the knowledge we use as ‘evidence’ can be understood as a type of epistemic injustice. The role of lived experience is evident in the development of intersectionality, where the founding thinkers, leading theorists, scholars and activists are black women in the US; a group which has faced, and continues to face, multiple levels of discrimination and oppression.^30,33,46^ Historically, academic ways of knowing have been dominated by elite, white men and this has affected the knowledge base and our ways of knowing.^33^ Epistemic injustice can result in insufficient conceptualisation of certain experiences within the collective ways of knowing, for example, if post-natal depression is not known in a culture, then the person experiencing this may individualise the experience.^51^ This justice issue is particularly relevant to the experience of chronic pain which is subjective in nature and relies on trust, belief and experience of the provider and collective knowledge base.^17^ Epistemic injustice is usually experienced with those holding less power and as such is often gendered and racialized.^17,51^

The concept of lived experience is of importance to both chronic pain research, and in contemporary health research methodology. Intersectionality offers a way of addressing the epistemic imbalance by centring the experience of the marginalised group/s both in selection of participants and methodology, also in considering the lived experience and positionality of our theorists. Racially minoritised women may offer more valuable insight to the nature of power precisely because they are more likely to experience oppression. Women, in particular women of colour, continue to apply intersectionality and call for its use in understanding inequities and power and to seek justice, including epistemic justice, for marginalised groups.^6,19,46^

## How can intersectionality inform research and knowledge creation?

### Epistemological and methodological considerations

Intersectionality can inform research epistemology (ways of knowing), priorities and methodology. In considering, for example, ‘Black Feminist Epistemology’, sociologist Patricia Hill Collins suggests that working class black women in the US may develop different priorities for research than those of more privileged groups.^33^ Developing and prioritising research questions with people who experience intersectional health inequities in chronic pain is one way that intersectionality could inform the epistemological and methodological foundations of research. There may also be deeper ontological questions, (about the nature of knowledge), and socially constructed concepts in chronic pain and care which benefit from an intersectional lens.

An intersectional approach can centre health research or evaluation on the experience of the marginalised group, rather than understanding the marginalised group in relation to the dominant group.^6,30,31^ There are several good examples of this in chronic pain research. Brady et al.^19,52^ worked with established community organisations to recruit and provide advocacy and interpretation during analysis for culturally and linguistically diverse communities in Sydney, Australia. Their intersectional analysis included gender, socioeconomic, ethnic and immigration status, with the aim of improving the knowledge of lived experience with chronic pain, framing the individual experience within Australian culture, and healthcare needs and access. Pryma^36^ provides a further example of this: to address the racialised gap in the knowledge around moral boundary work for women with fibromyalgia in the US, she used intersectionality to frame the study analysis to include ‘race’, gender and class. In the UK, Bull et al.^1^ provide an example of centring the analysis of their pain management group evaluation on patients from racially minoritised groups, including intersections of disabled status, faith and immigration status.

Intersectional and antiracist frameworks centre the experiences of marginalised groups, underpinned by social justice. The choice of a method to meet these needs could include community based participatory research and participatory action research methods.^6,31,47^ Research might include small scale, grassroots, community projects of typically overlooked populations.^30^ In this process, researchers are acknowledged as social actors and reflexivity is an important part of the methodology.^6,30^ Researchers can consider their own social identities in an intersectional way and the implications of their own experiences on the research, as per our previous example.^13^ Using reflexivity and positionality in this way can be used in both qualitative and quantitative research.^27,47^

Intersectionality has been conceptualised, and implemented, in different ways in literature reviews. De Jong et al.^53^ used intersectionality as a framework during a scoping review to develop analysis and policy recommendations for foetal alcohol spectrum disorder in South Africa, framing intersecting oppressions from downstream individual factors to upstream factors including the colonial context, racism and policy history. Husain et al.^54^ hoped to select studies that used intersectionality as a testable theory on the impact of marginalisation across intersecting categories of demographics with regards to digital care, but found that the studies used descriptive, rather than explanatory analysis. Collins^30^ states that intersectionality is not a typical, testable theory. However, Husain et al.^54^ built on previous work of McCall^55^ from a quantitative, big data background, where McCall notes sacrifices to the conceptualisation of multiple intersecting oppressions in her approach. Kapilashrami et al.^35^ draw attention to the fluid nature of categories of social identity and the contextual nature of interactions and maintenance in systems of power; they need to be understood as more than the sum of their parts.

## Data collection

Data should be collected in a sensitive way, and the relevance of the local context and the multitude of factors that may influence this should be considered.^6^ Language, categorisation and their associated meanings could help or hinder engagement of different groups. People from marginalised groups could guide the terminology and methods for involvement of participants. Terminology around concepts such as ‘working class’, ‘difficulty making ends meet’ and ‘socioeconomic deprivation’; ‘disabled’ or in receipt of ‘disability benefits’; ‘ethnic minority groups’ or ‘racialised groups’; and LGBTQIA + identification would benefit from input with relevant groups and knowledgeable about the local context, in addition to consideration of approaches other researchers have used.^26,36,56,57^ Following recruitment, data collection of demographics/social identities may be complex. Brady et al.^19,52^ offer a good example of brief initial interviews that were undertaken to collect intersectional data. The FOR-EQUITY website^58^ offers a range of tools that may be helpful in data collection and research conceptualisation.

An intersectional lens can be applied to quantitative data collection, and this has been used descriptively.^54^ Assumptions about both chronic pain and the highly contextual nature of intersectional health inequities should be made clear, for example, the experience and stress associated with racism will vary considerably across ethnic groups, country and with migration status. Demographic data should include ethnicity, gender, socioeconomic measures and be put into geographical context.^9^ Study outcomes could be understood in the context of demographics and socio-political context, helping to better understand who does and does not benefit from interventions across population groups and highlight areas for improvement along equity lines. Following their wide review of health inequities in Scotland, Miall et al.^59^ advocate intersectionality as a useful lens to provide better understanding of the complexity associated with the multiple axes of health inequities. If used this way, it is important to understand any quantitative data or biological impacts within the context of the social world, and as previously discussed, there may be trade-offs in conceptualisation of intersecting oppressions.^55^ When interpreting experiences of racism and how the distress associated with this may impact on biological measures, it is important to frame the subject of change as racism rather than race.^48^

## Trial inclusion

Intersectionality can provide more theory and grounding to inclusive approaches to research methods. However, it is important to be mindful of historical legacies of health inequities in the knowledge base prior to the trial stage. As outlined earlier, research processes can be exclusionary and require work to be more inclusive.^48,60^ To be more representative of the populations they intend to benefit, trials would require to adapt methodology, which may take more time, effort and money and this includes the development of relationships with different communities.^61,62^ Recently, trial methodology has improved to become more inclusive of those impacted by health inequities, meaning research can better meet the needs of the populations who it should serve.^60,61^ For instance, Rai et al.^56^ adapted their study design to foster greater inclusion of racially minoritised participants. They found that being more inclusive takes more time, and therefore money, which makes inclusion more challenging in a short-term research grant frame. In the U.K., the INCLUDE project has been established by the National Institute for Health and Care Research (NIHR) to help trials be more representative of the populations they intend to serve, which is expected to deliver better quality, applicability and credibility of health research data, thus leading to better healthcare.^62^ In this context, ‘representativeness’ may be taken as related to the population of the U.K. With regard to inclusion, the term ‘inclusive research’ is often used in the literature; however, a worthwhile consideration is the idea of doing research inclusively, bringing the ongoing ethos and approach of *being inclusive*.^63^ If considering representation, intersectionality may help by drawing attention to the multiple demographic categories, however, intersectionality is essentially about understanding the nature of the multiple, interconnected systems of power and oppression that maintain inequities, as illustrated in Figure 1.

## Patient and public involvement and engagement

We have expressed the importance of lived experience in the development of knowledge. An increasingly recognised way of embedding lived experience to the benefit of research is through Patient and Public Involvement and Engagement (PPIE), from conception of research, including setting research priorities, to dissemination of research.^64,65^ Roles and tasks can include being co-applicants on grants, part of an advisory group, giving feedback on paperwork, and agreement should be made about the purpose of PPIE and any training needs, which can depend on both the needs of the research and preferences of the people involved.^64,65^ PPIE could also be considered in an intersectional way, in that there are multiple lived experiences of health phenomena, and we should be mindful of our own positionality and power as researchers, and that our experiences may be quite different to those of our PPIE colleagues. Without aims of social change, research which involves lived experience can be viewed as oppressive and therefore the purpose of research should be considered.^66^

Approaches to PPIE could benefit from the Participatory Action Research (PAR) literature, which promotes health equity and fosters approaches to include marginalised groups who, as part of the cultural, systematic devaluing of lived experience as a form of knowledge, may undervalue their own lived knowledge.^67,68^ We make the case here for a theoretical underpinning to research with which we can attend to the way our knowledge is created, and therefore PAR offers a helpful example of methodology underpinned by equitable principles applied to knowledge construction. Belton et al.^69^ also argue that lived experience should play a key role in improving health systems and should be underpinned by equitable principles and methods to foster inclusion of marginalised groups. Fostering inclusion, equity and relational approaches could also help to counter the ‘elite capture’, which can otherwise occur within PPIE.^66^ Further consideration could be given to common barriers to participation in research, likely similar to barriers in involvement in PPIE, and include travel, time and literacy.^62^ Barriers should be viewed systemically, including limited funding, short-term contracts, tight deadlines, negotiating normative codes and power structures in academia.^66^

The NIHR^65^ offer helpful guidance on PPIE, which also covers compensation for time and interaction of this with the U.K. social security system, and organisational responsibilities. PPIE is not in itself equitable and, without care, could further approaches focused on individualism, particularly in a neoliberal care and research environment. We must therefore take care to underpin our approaches to PPIE in equitable principles, and we suggest intersectionality as a framework for informing this approach.

## Future directions and conclusions

We draw attention to the contextual limitations of this paper as outlined in the introduction and suggest scope to develop intersectionality as a theoretical framework in pain research to include indigenous populations in countries colonised by Europeans, and intersectional axes of health inequities beyond high-income countries. We have illustrated six intersecting axes of health inequities which represent significant causes of oppression faced by people in the U.K. and are supported in the literature, in Figure 1. However, in this paper, we focused on the core intersections of gender, race and class, with further evidence of the impact of ethno-nationalism. We have noted ableism and queer-phobia as oppressions but have not detailed further specifics on this related to pain. These are current areas of significant concern for liberty, and therefore health, in the U.K. and we advocate not only further research, but action in opposition to suppression of liberties. The creation of inclusive and equitable approaches to pain research and care can, unfortunately, be countered by government rhetoric and policies that exacerbate the marginalisation of groups (for example, asylum seekers and transgender people), and austerity policies.^70^ The increasingly authoritarian nature of government in the U.K. is of further concern on this point.^71^ We must not only be more active in our role as researchers and practitioners, but also as activists and advocates. For instance, we could be more critical of the political implications of knowledge creation and use, and the role of ideologies such as neoliberalism and capitalism in shaping health, care and research, as these favour individualism and competitive research design and delivery systems that focus on short time frames and linear perspectives.

By using intersectionality as a theoretical framework, we are better able to embrace the necessary complexity for researching chronic pain, and pain care, equitably. The use of a reductionist frame can force unachievable objectivity on to complex health concepts, and there is an increasing realisation in the field that we need to understand the individual within the social world,^26^ hence the intersectional perspective of social identities conceptualised with associated oppressions. We must further recognise that each step of the research process is more subjective than we often think^48^ and, by overstating claims of objectivity, we put at risk our ability to make a difference in the real world and to the people who need both research and clinical focus the most. Intersectionality offers a framework that takes account of multiple, simultaneously held, collective identities that has implications for each aspect of research and privileges socially constructed meaning.

## References

[1] Bull E Young D Etchebarne A , et al. Understanding ethnic minority service user experiences of being invited to and attending group pain programmes: a qualitative service evaluation. Br J Pain 2023; 17: 58–70. 36815070 10.1177/20494637221129196 PMC9940249

[2] Moore FR Williams L Dunbar M . Sociodemographic predictors of attendance at a Scottish pain management programme. Br J Pain 2021; 15: 393–400. 34840787 10.1177/2049463720970579 PMC8611294

[3] McCartney G Popham F McMaster R , et al. Defining health and health inequalities. Public Health 2019; 172: 22–30. 31154234 10.1016/j.puhe.2019.03.023 PMC6558275

[4] McCartney G Dickie E Escobar O , et al. Health inequalities, fundamental causes and power: towards the practice of good theory. Sociol Health Illn 2021; 43: 20–39. 33222244 10.1111/1467-9566.13181 PMC7894306

[5] Lucyk K McLaren L . Taking stock of the social determinants of health: a scoping review. PLoS One 2017; 12: e0177306. 28493934 10.1371/journal.pone.0177306 PMC5426664

[6] Heard E Fitzgerald L Wigginton B , et al. Applying intersectionality theory in health promotion research and practice. Health Promot Int 2020; 35: 866–876. 31390472 10.1093/heapro/daz080

[7] Pickett KE Wilkinson RG . Income inequality and health: a causal review. Soc Sci Med 2015; 128: 316–326. 25577953 10.1016/j.socscimed.2014.12.031

[8] GBD 2017 Disease and Injury Incidence and Prevalence Collaborators . Global, regional, and national incidence, prevalence, and years lived with disability for 354 diseases and injuries for 195 countries and territories, 1990-2017: a systematic analysis for the Global Burden of Disease Study 2017 GBD 2017 Disease and Injury Incidence and Prevalence Collaborators. Lancet 2018; 392: 1789–1858. 30496104 10.1016/S0140-6736(18)32279-7 PMC6227754

[9] Welch V Petticrew M Tugwell P , et al. PRISMA-Equity 2012 extension: reporting guidelines for systematic reviews with a focus on health equity. PLoS Med 2012; 9: e1001333. 23222917 10.1371/journal.pmed.1001333 PMC3484052

[10] Fink AM . Toward a new definition of health disparity: a concept analysis. J Transcult Nurs 2009; 20: 349–357. 19581385 10.1177/1043659609340802

[11] Dickie E Hearty W Fraser A , et al. Power – a health and social justice issue. Scotland, UK: NHS Health Scotland, 2015.

[12] Foucault M . Power: essential works 1954-84. London: Penguin Random House, 2020.

[13] Jacobson D Mustafa N . Social identity map: a reflexivity tool for practicing explicit positionality in critical qualitative research. Int J Qual Methods 2019; 18: 160940691987007.

[14] Treede R Rief W Barke A , et al. Chronic pain as a symptom or a disease: the IASP classification of chronic pain for the international classification of diseases (ICD-11). Pain 2019; 160: 19–27. 30586067 10.1097/j.pain.0000000000001384

[15] Nicholas M Vlaeyen JWS Rief W , et al. The IASP classification of chronic pain for ICD-11: chronic primary pain. Pain 2019; 160: 28–37. 30586068 10.1097/j.pain.0000000000001390

[16] Medicine and Society . Chronic pain patients and the biomedical model of pain. Virtual Mentor - American Medical Association Journal of Ethics 2013; 15: 455–459. 23680569 10.1001/virtualmentor.2013.15.5.msoc1-1305

[17] Buchman DZ Ho A Goldberg DS . Investigating trust, expertise, and epistemic injustice in chronic pain. J Bioeth Inq 2017; 14: 31–42. 28005251 10.1007/s11673-016-9761-x

[18] Zajacova A Grol-Prokopczyk H Zimmer Z . Sociology of chronic pain. J Health Soc Behav 2021; 62: 302–317. 34283649 10.1177/00221465211025962 PMC8956223

[19] Brady B Veljanova I Chipchase L . The intersections of chronic noncancer pain: culturally diverse perspectives on disease burden. Pain Med 2019; 20: 434–445. 29846709 10.1093/pm/pny088

[20] White K. An introduction to the sociology of health and illness. Second edition. London, UK: Sage, 2011.

[21] Allen J Balfour R Bell R , et al. Social determinants of mental health. Int Rev Psychiatry 2014; 26: 392–407. 25137105 10.3109/09540261.2014.928270

[22] Nicholas MK Linton SJ Watson PJ , et al. Early identification and management of psychological risk factors (“yellow flags”) in patients with low back pain: a reappraisal. Phys Ther 2011; 91: 737–753. 21451099 10.2522/ptj.20100224

[23] Linton SJ Shaw WS . Impact of psychological factors in the experience of pain. Phys Ther 2011; 91: 700–711. 21451097 10.2522/ptj.20100330

[24] Chapman CR Tuckett RP Song CW . Pain and stress in a systems perspective: reciprocal neural, endocrine, and immune interactions. J Pain 2008; 9: 122–145. 18088561 10.1016/j.jpain.2007.09.006 PMC2278005

[25] Jones GT Power C Macfarlane GJ . Adverse events in childhood and chronic widespread pain in adult life: results from the 1958 British Birth Cohort Study. Pain 2009; 143: 92–96. 19304391 10.1016/j.pain.2009.02.003

[26] Webster F Connoy L Sud A , et al. Chronic struggle: an institutional ethnography of chronic pain and marginalization. J Pain 2023; 24: 437–448. 36252618 10.1016/j.jpain.2022.10.004

[27] Morais CA Aroke EN Letzen JE , et al. Confronting racism in pain research: a call to action. J Pain 2022; 23: 878–892. 35292201 10.1016/j.jpain.2022.01.009 PMC9472374

[28] May CR Eton DT Boehmer K , et al. Rethinking the patient: using burden of treatment theory to understand the changing dynamics of illness. BMC Health Serv Res 2014; 14: 281. 24969758 10.1186/1472-6963-14-281 PMC4080515

[29] Corbin J Strauss A . Managing chronic illness at home: three lines of work. Qual Sociol 1985; 8: 224–247.

[30] Collins PH . Intersectionality’s definitional dilemmas. Annu Rev Sociol 2015; 41: 1–20.

[31] Bowleg L . The problem with the phrase women and minorities: intersectionality – an important theoretical framework for public health. Am J Public Health 2012; 102: 1267–1273. 22594719 10.2105/AJPH.2012.300750 PMC3477987

[32] Davis AY . Women, race and class. New York, NY: Penguin, 1981.

[33] Collins PH . Black feminist thought. Second Edition. London, UK: Routledge, 2009.

[34] Crenshaw K . Demarginalizing the intersection of race and sex: a Black feminist critique of antidiscrimination doctrine, feminist theory and antiracist politics. Chicago, IL: Univ Chicago Leg Forum, 1989, http://chicagounbound.uchicago.edu/uclf/vol1989/iss1/8

[35] Kapilashrami A Hill S Meer N . What can health inequalities researchers learn from an intersectionality perspective? Understanding social dynamics with an inter-categorical approach? Soc Theory Health 2015; 13: 288–307.

[36] Pryma J. “Even my sister says I’m acting like a crazy to get a check”: race, gender, and moral boundary-work in women’s claims of disabling chronic pain. Soc Sci Med 2017; 181: 66–73. 28376357 10.1016/j.socscimed.2017.03.048

[37] Varpio L Paradis E Uijtdehaage S , et al. The distinctions between theory, theoretical framework, and conceptual framework. Acad Med 2020; 95: 989–994. 31725464 10.1097/ACM.0000000000003075

[38] Hathcoat JD Meixner C Nicholas MC . Ontology and epistemology. In: Liamputtong P (ed). Handbook of research methods in health social sciences. Singapore: Springer, 2019.

[39] Rees C Crampton P Monrouxe L . Re-visioning academic medicine through a constructionist lens. Acad Med 2020; 95: 846–850. 31809294 10.1097/ACM.0000000000003109

[40] Paradis E Nimmon L Wondimagegn D , et al. Critical theory: broadening our thinking to explore the structural factors at play in health professions education. Acad Med 2020; 95: 842–845. 31809292 10.1097/ACM.0000000000003108

[41] Macgregor C Walumbe J . We need to develop our approach to socially constructed concepts including socioeconomic factors, power, ethnicity and racism in pain care and research. Pain and Rehabilitation 2021; 51: 1–4.

[42] Webster F Connoy L Longo R , et al. Patient responses to the term pain catastrophizing: thematic analysis of cross-sectional international data. J Pain 2023; 24: 356–367. 36241160 10.1016/j.jpain.2022.10.001 PMC9898136

[43] National Institute for Clinical Excellence . Chronic pain (primary and secondary) in over 16s: assessment of all chronic pain and management of chronic primary pain. London, UK: National Institute for Clinical Excellence, 2021. 33939353

[44] Burton AE Shaw RL . Pain management programmes for non-English-speaking black and minority ethnic groups with long-term or chronic pain. Musculoskeletal Care 2015; 13: 187–203. 25784618 10.1002/msc.1099

[45] Ashar YK Gordon A Schubiner H , et al. Effect of pain reprocessing therapy vs placebo and usual care for patients with chronic back pain: a randomized clinical trial. JAMA Psychiatry 2022; 79: 13–23. 34586357 10.1001/jamapsychiatry.2021.2669 PMC8482298

[46] Davis AY Dent G Meiners EM , et al. Abolition. Feminism. Now. New York, NY: Penguin Random House, 2022.

[47] Hood AM Booker SQ Morais CA , et al. Confronting racism in all forms of pain research: a shared commitment for engagement, diversity, and dissemination. J Pain 2022; 23: 913–928. 35288029 10.1016/j.jpain.2022.01.008 PMC9415432

[48] Letzen JE Mathur VA Janevic MR , et al. Confronting Racism in all forms of pain research: reframing study designs. J Pain 2022; 23: 893–912. 35296390 10.1016/j.jpain.2022.01.010 PMC9472383

[49] Wailoo K . Pain: a political history. Baltimore, US: Johns Hopkins University Press, 2014.

[50] Fioramonti L Coscieme L Costanza R , et al. Wellbeing economy: an effective paradigm to mainstream post-growth policies? Ecological Economics 2022; 192: 107261.

[51] Fricker M . Epistemic injustice: power and the ethics of knowing. Oxford, UK: Oxford University Press, 2007.

[52] Brady B Veljanova I Chipchase L . An exploration of the experience of pain among culturally diverse migrant communities. Rheumatol Adv Pract 2017; 1: rkx002. 31431942 10.1093/rap/rkx002 PMC6649908

[53] De Jong M George A Jacobs T . A scoping review of the determinants of foetal alcohol spectrum disorder in South Africa: an intersectional perspective. Health Policy Plan 2021; 36: 1459–1469. 34508361 10.1093/heapol/czab101 PMC8505989

[54] Husain L Greenhalgh T Hughes G , et al. Desperately seeking intersectionality in digital health disparity research: narrative review to inform a richer theorization of multiple disadvantage. J Med Internet Res 2022; 24: e42358. 36383632 10.2196/42358 PMC9773024

[55] McCall L . The complexity of intersectionality. Signs: Journal of Women in Culture and Society 2005; 30: 1771–1800.

[56] Rai T Hinton L McManus RJ , et al. What would it take to meaningfully attend to ethnicity and race in health research? Learning from a trial intervention development study. Sociol Health Illn 2022; 44: 57–72. 35023187 10.1111/1467-9566.13431 PMC10078726

[57] Zhang B Chang B Du W . Employing intersectionality as a data generation tool: suggestions for qualitative researchers on conducting interviews of intersectionality study. Int J Qual Methods 2021; 20: 160940692110646.

[58] For-Equity , FOR-EQUITY website. 2022. https://forequity.uk (Accessed on 24/3/2022)

[59] Miall N Fergie G Pearce A . Health Inequalities in Scotland: trends in deaths, health and wellbeing, health behaviours, and health services since 2000. Glasgow, Scotland: University of Glasgow, 2022

[60] The National Institute for Health Research . Improving inclusion of under-served groups in clinical research: guidance from include project. Bethesda, MA: National Institute for Health Research, 2020. Available at: https://www.nihr.ac.uk/documents/improving-inclusion-of-under-served-groups-in-clinical-research-guidance-from-include-project/25435 (Accessed 11 November 2022)

[61] Dawson S Banister K Biggs K , et al. Trial Forge Guidance 3: randomised trials and how to recruit and retain individuals from ethnic minority groups—practical guidance to support better practice. Trials 2022; 23: 672. 35978338 10.1186/s13063-022-06553-w PMC9383663

[62] Witham MD Anderson E Carroll C , et al. Developing a roadmap to improve trial delivery for under-served groups: results from a UK multi-stakeholder process. Trials 2020; 21: 694. 32738919 10.1186/s13063-020-04613-7 PMC7395975

[63] Nind M Kaley A Hall E . Focus Group Method. In: Liamputtong P (ed). Handbook of social inclusion. Berlin, Germany: Springer International Publishing, 2022.

[64] Hoens AM Belton J Scott A , et al. Patients as partners in research: there is plenty of help for researchers. J Orthop Sports Phys Ther 2020; 50: 219–221. 32354313 10.2519/jospt.2020.0104

[65] The National Institute for Health Research . Briefing notes for researchers - public involvement in NHS, health and social care research. Bethesda, MA: National Institute for Health Research, 2021. Available at: https://www.nihr.ac.uk/documents/briefing-notes-for-researchers-public-involvement-in-nhs-health-and-social-care-research/27371 (Accessed 24 May 2021).

[66] Okoroji C Mackay T Robotham D , et al. Epistemic injustice and mental health research: a pragmatic approach to working with lived experience expertise. Front Psychiatry 2023; 14: 1114725. 37056406 10.3389/fpsyt.2023.1114725 PMC10086175

[67] Lawson HA Caringi J Pyles L et al. Participatory action research. Oxford, UK: Oxford University Press, Inc., 2015.

[68] Baum F Macdougall C . Participatory action research. J Epidemiol Community Health 2006; 60: p854–p857. 10.1136/jech.2004.028662 PMC2566051 16973531

[69] Belton JL Slater H Ravindran TKS , et al. Harnessing people’s lived experience to strengthen health systems and support equitable musculoskeletal health care. J Orthop Sports Phys Ther 2023; 53: p1–p10. 10.2519/jospt.2022.11427 36507691

[70] Walsh D Dundas R Mccartney G , et al. Bearing the burden of austerity: how do changing mortality rates in the UK compare between men and women? J Epidemiol Community Health 2022; 76: 1027–1033. 36195463 10.1136/jech-2022-219645 PMC9664129

[71] Butler P . ‘Hostile, authoritarian’ UK downgraded in civic freedoms index. London, UK: The Guardian, 2023.

[72] Prego-Domínguez J Khazaeipour Z Mallah N , et al. Socioeconomic status and occurrence of chronic pain: a meta-analysis. Rheumatol 2021; 60: 1091–1105. 10.1093/rheumatology/keaa758 33276382

[73] Public Health England . Chronic pain in adults 2017. Health survey for England. London, UK: Department of Health and Social Care, UK Government 2020.

[74] Mordecai L Reynolds C Donaldson LJ , et al. Patterns of regional variation of opioid prescribing in primary care in England: a retrospective observational study. Br J Gen Pract 2018; 68: e225–e233. 29440012 10.3399/bjgp18X695057 PMC5819988

[75] Torrance N Mansoor R Wang H , et al. Association of opioid prescribing practices with chronic pain and benzodiazepine co-prescription: a primary care data linkage study. Br J Anaesth 2018; 120: 1345–1355. 29793600 10.1016/j.bja.2018.02.022

[76] Jain S Jadwani V Spogmy S , et al. Inequalities and inequities in the types of chronic pain services available in areas of differing deprivation across England. Scand J Pain 2023; 23: 168–174. 35503225 10.1515/sjpain-2022-0015

[77] Fisher R Dunn P Asaria M , et al. Level or not? Comparing general practice in areas of high and low socioeconomic deprivation in England. London, UK: The Health Foundation, 2020, https://www.health.org.uk/publications/reports/level-or-not

[78] McLean G Gunn J Wyke S , et al. The influence of socioeconomic deprivation on multimorbidity at different ages: a cross-sectional study. Br J Gen Pract 2014; 64: e440–e447. 24982497 10.3399/bjgp14X680545 PMC4073730

[79] Fayaz A Croft P Langford RM , et al. Prevalence of chronic pain in the UK: a systematic review and meta-analysis of population studies. BMJ Open 2016; 6: e010364. 10.1136/bmjopen-2015-010364 PMC4932255 27324708

[80] Keogh E . The gender context of pain. Health Psychol Rev 2021; 15: 454–481. 32875959 10.1080/17437199.2020.1813602

[81] Nicholl BI Smith DJ Cullen B , et al. Ethnic differences in the association between depression and chronic pain: cross sectional results from UK Biobank. BMC Fam Pract 2015; 16: 128. 26445480 10.1186/s12875-015-0343-5 PMC4596418

[82] Haywood C Tanabe P Naik R , et al. The impact of race and disease on sickle cell patient wait times in the emergency department. Am J Emerg Med 2013; 31: 651–656. 23380119 10.1016/j.ajem.2012.11.005 PMC3608692

[83] Linton EA Goodin DA Hankins JS , et al. A survey-based needs assessment of barriers to optimal sickle cell disease care in the emergency department. Ann Emerg Med 2020; 76: S64–S72. 32928465 10.1016/j.annemergmed.2020.08.013 PMC7511000

[84] Lamotte JE Hills GD Henry K , et al. Understanding the roots of mistrust in medicine: learning from the example of sickle cell disease. J Hosp Med 2022; 17: 495–498. 35535934 10.1002/jhm.12800 PMC9303871

[85] Power-Hays A McGann PT . When actions speak louder than words — racism and sickle cell disease. N Engl J Med 2020; 383: 1902–1903. 32871062 10.1056/NEJMp2022125

